# Photovoltaic Ge/Si quantum dot detectors operating in the mid-wave atmospheric window (3 to 5 *μ*m)

**DOI:** 10.1186/1556-276X-7-494

**Published:** 2012-08-31

**Authors:** Andrew Yakimov, Vyacheslav Timofeev, Aleksei Bloshkin, Aleksandr Nikiforov, Anatolii Dvurechenskii

**Affiliations:** 1 Rzhanov Institute of Semiconductor Physics, Siberian Branch of the Russian Academy of Sciences, Prospekt Lavrent’eva 13, Novosibirsk, 630090, Russia

**Keywords:** Quantum dots, Silicon, Germanium, Interband transitions, Infrared photodetectors

## Abstract

Ge/Si quantum dots fabricated by molecular-beam epitaxy at 500°C are overgrown with Si at different temperatures *T*_cap_, and effect of boron delta doping of Si barriers on the mid-infrared photoresponse was investigated. The photocurrent maximum shifts from 2.3 to 3.9 *μ*m with increasing *T*_cap_from 300°C to 750°C. Within the sample set, we examined devices with different positions of the *δ*-doping layer with respect to the dot plane, different distances between the *δ*-doping layer and the dot plane *d*, and different doping densities *p*_B_. All detectors show pronounced photovoltaic behavior implying the presence of an internal inversion asymmetry due to the placing dopants in the barriers. The best performance was achieved for the device with *T*_cap _= 600°C, *p*_B _= 12 × 10^11^cm^−2^, and *d *= 5 nm in a photovoltaic regime. At a sample temperature of 90 K and no applied bias, a responsivity of 0.83 mA/W and detectivity of 8 × 10^10^ cm Hz^1/2^/W at *λ *= 3.4 *μ*m were measured under normal incidence infrared radiation.

## Background

In the past years, there has been a surge of interest in structures that exhibit quantum confinement in all three dimensions, commonly known as quantum dots (QDs). Intersubband optical transitions in QDs have attracted a great deal of attention due to their potential applications in infrared detectors operating at normal incidence and displaying low dark current [1,2]. Most of the demonstrations of quantum dot infrared photodetectors (QDIPs) were achieved with III to V heterostuctures. Ge QDs enclosed in a silicon matrix represent another attractive type of the device due to its compatibility with standard Si readout circuitry. In particular, it has been demonstrated that *p*-type Ge/Si(001) QDs exhibit intraband photoresponse in the spectral range of 3 to 5 *μ*m [3-7], thus opening the route towards the fabrication of Si-based QDIPs for mid-infrared atmospheric window. Photovoltaic sensors operating without external bias voltage are preferrable for application in focal plane arrays as they have the advantage of reduced noise equivalent temperature difference [8]. In this paper, performance of ten-period Ge/Si quantum-dot mid-infrared photodetectors was investigated. By adjusting the capping temperature and Si delta-doping parameters, we demonstrated a responsivity of 0.83 mA/W and detectivity of 8 × 10^10^ cm Hz^1/2^/W at photon wavelength (*λ*) = 3.4 *μ*m for a Ge/Si device operating in a photovoltaic mode.

## Methods

The samples were grown by solid source molecular beam epitaxy on a (001)-oriented boron-doped *p*^+ ^-Si substrate with resistivity of 0.01 *Ω* cm. An active region was composed of ten stacks of Ge quantum dots separated by 50-nm Si barriers. Each Ge QD layer consisted of a nominal Ge thickness of about 6 monolayers (ML) and formed by self-assembling in the Stranski-Krastanov growth mode at 500°C and at a growth rate of 0.2 ML/s for all samples. From scanning tunneling microscopy experiments with uncapped samples, we observed the Ge dots to be approximately 10 to 15 nm in lateral size and about 1.0 to 1.5 nm in height. They have the form of hut clusters bounded by {105} facets. The density of the dots is about 3 to 4 × 10^11^ cm^−2^. Immediately after the deposition of Ge, the temperature was lowered to 400°C, and the structure was covered by a 1-nm Si layer. This procedure is necessary to preserve island shape from the effect of a further high temperature overgrowth. The Si barriers were deposited at *T*_cap_ ranging from 300°C to 750°C for different samples, with temperature ramps before and after QD growth. The increase of the overgrowth temperature is expected to result in a lowering of the hole binding energy in the dots due to enhanced Si-Ge alloying [9].

The active region was sandwiched in between the 200-nm-thick intrinsic Si buffer and cap layers. Finally, a 200-nm-thick *p*^+ ^-Si top contact layer (5 × 10^18^cm^−3^) was deposited. For vertical photocurrent measurements, the samples were processed in the form of circular mesas with diameter of 3 mm by wet chemical etching and contacted by Al/Si metallization. The bottom contact is defined as the ground when applying voltage to all detectors.

Each Si barrier contains a boron delta-doping layer located near the QD plane to provide holes to the dots. To study the effects of doping on the detector characteristics, we varied (a) the relative position of the *δ*-doping layer with respect to the QD plane, (b) the distance between the *δ*-doping layer and the QD plane, and (c) the doping level. In a first type of devices, referred to as devices with bottom doping, the delta-doping layer with a sheet hole density of *p*_B _= 8 × 10^11^cm^−2 ^was inserted 5 nm below the Ge QD layer to yield about two holes per dot. In a second type of detectors, the delta-doping layer was placed above the Ge dots with boron concentration *p*_B _= 4 × 10^11^cm^−2^ (about one hole per QD), 8 × 10^11^cm^−2^ (approximately two holes per QD), and 12 × 10^11^cm^−2^(three to four holes per QD). The distance between the dots and the doping plane was *d *= 2, 5, and 10 nm.

The normal-incidence photoresponse was obtained using a Bruker Vertex 70 Fourier transform infrared spectrometer (Bruker Optik Gmbh, Ettlingen, Germany) with a spectral resolution of 5 cm^−1 ^along with a SR570 low-noise current preamplifier (Stanford Research Systems, Inc., Sunnyvale, CA, USA). The photocurrent (PC) spectra were calibrated with a DLaTGS detector (SELEX Galileo Inc., Arlington, VA, USA). The noise characteristics were measured with an SR770 fast Fourier transform analyzer (Stanford Research Systems, Inc.), and the white noise region of the spectra was used to determine the detectivity. The sample noise was obtained by subtracting the preamplifier-limited noise level from the experimental data. The dark current was tested as a function of bias (*U*_*b*_) by a Keithley 6430 Sub-Femtoamp Remote SourceMeter (Keithley Instruments Inc., Cleveland, OH, USA). The devices were mounted in a cold finger inside a Specac cryostat with ZnSe windows. For dark current and noise measurements, the samples were surrounded with a cold shield.

## Results and discussion

**Figure 1:**
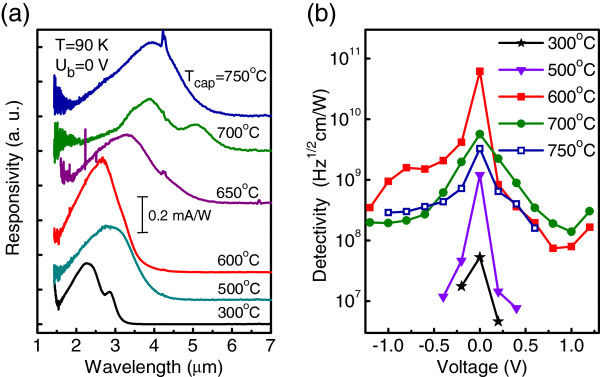
**Photocurrent spectra and specific detectivity.** Of Ge/Si QD samples where the capping temperature *T*_cap _was systematically varied. (**a**) Series of responsivity spectra measured at temperature of 90 K with no applied bias (*U*_*b *_= 0 V). The spectra have been vertically displaced for clarity. (**b**) Detectivity as a function of applied bias. The measurement temperature is 90 K. For the samples with *T*_cap _= 300°C, 500°C, and 600°C, the data were taken at *λ *= 3 *μ*m. For the samples with *T*_cap _= 700°C and 750°C, the peak detectivity is shown.

Figure 1a depicts the photovoltaic spectral response at *T *= 90 K from six detectors with bottom doping (*p*_B _= 8 × 10^11^ cm^−2^, *d *= 5 nm) in which the overgrowth temperature was varied from 300°C to 750°C. In all samples, a broad peak is observed with no applied voltage. As expected, the peak wavelength of the device shows a redshift from 2.3 to 3.9 *μ*m with increasing _*T*cap_. This is significant since it provides a recipe to control the operating wavelength of a Ge/Si QD detector. The specific detectivity is given by $D^{*}=R_{s}\sqrt{A\cdot \mathrm{\Delta f}}/i_{n}$, where *A* is the device area; *R*_*s*_, the responsivity; *i*_*n*_, the noise current; and *Δf*, the bandwidth. The detectivity obtained from the devices at 90 K is shown in Figure 1b. Despite the responsivity value is approximately the same for all samples, the detectivity is ultimate for *T*_cap _= 600°C. The physical mechanism of such a behavior is still under investigation. We suspect that at low cap temperature, a large number of point defects are generated in Si layers producing high noise level [10]. At the same time, at *T*_cap _> 700°C, the structure resembles a two-dimensional SiGe layer with significant composition and thickness variations [9], thus, again giving rise to a large dark current.

In fact, capping of Ge islands under a Si layer at elevated *T*_cap _results both in a reduction of Ge content in the dots and growth of dot size. The former implies a lowering of the hole binding energy due to the decrease of the valence band offset, while the latter leads to a deepening of the hole level due to the weakening of the size quantization effect. The competition of these two processes is a possible reason of why the peak wavelength shows a nonmonotonic shift with *T*_cap_ around 600°C as observed in Figure 1a.

**Figure 2:**
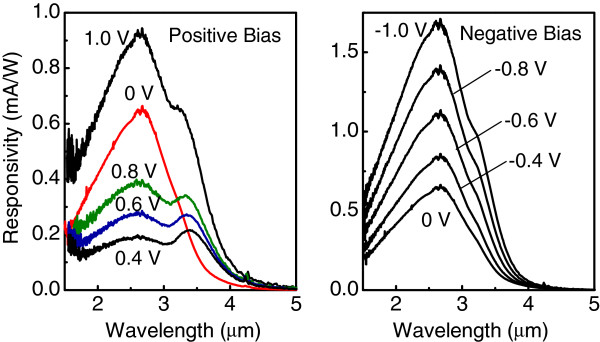
**Responsivity spectra under different applied bias of QDIP with the bottom position of the delta-doping plane.** The distance between the QD layer and the doping plane is 5 nm; the sheet hole density *p*_B _= 8 × 10^11^cm^−2^. The sample temperature is 90 K.

**Figure 3:**
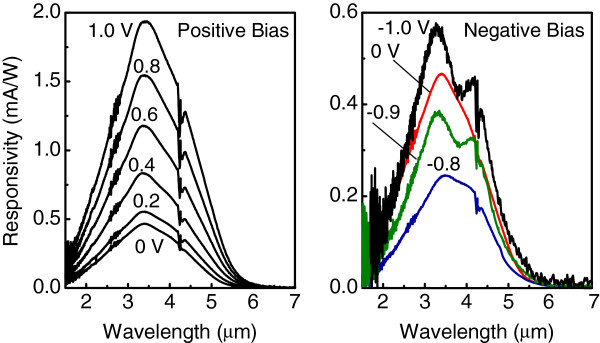
**Responsivity spectra under different applied bias of QDIP with the top position of the delta-doping plane.** The distance between the QD layer and the doping plane is 5 nm; the sheet hole density *p*_B _= 8 × 10^11^cm^−2^. The sample temperature is 90 K.

Figures 2 and 3 show bias-dependent PC spectra of the devices with bottom and top positions of the delta-doping plane, respectively. In both samples, the distance between the QD layer and the doping plane is 5 nm, the sheet hole density *p*_B _= 8 × 10^11^cm^−2^. A broad mid-infrared peak is observed with no applied bias implying the presence of a built-in electric field near the dots. An applied external bias (positive in Figure 2 and negative in Figure 3) compensates the internal electric field, thus leading to a drastic drop of responsivity at 0.4 to 0.8 V in Figure 2 and around -0.8 V in Figure 3. This is a characteristic feature of a photovoltaic mode operation [11]. At large *U*_*b*_, the photoexcited holes can easily overcome the built-in potential barrier, and responsivity starts to increase.

**Figure 4:**
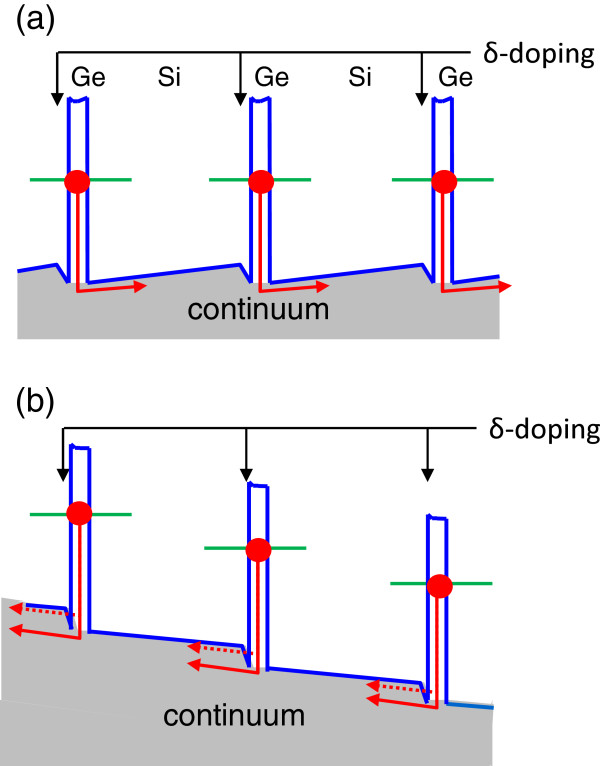
**Valence band diagram with the involved hole transitions in the QDIP.** With delta-doping planes for (**a**) zero bias and (**b**) *U*_*b *_≠ 0. The closed circles show the states occupied with a hole. The arrows indicate the hole flow.

The interesting feature is the observation of two distinct peaks at a positive bias for the sample with bottom doping and at a negative bias for the sample with top doping. An additional photoresponse at a longer wavelength appears when the applied bias polarity is such that holes escape from the QDs towards the nearest *δ*-doping plane. The origin of photovoltaic signal and dual-peak response is illustrated in Figure 4. This is caused by asymmetry of the samples due to selected position of the doping planes within the spacer layers between the dots. At zero bias, the resulting potential profile leads to a preferential hole flow in the direction opposite to the nearest *δ*-doping layer side (Figure 4a). The corresponding PC peak arises from the transition of holes from the ground state to the continuum states. When the polarity of applied bias *U*_*b *_is that holes move toward the doping layer, holes can be transferred into the extended state by excitation to a shallow level confined near the valence band edge with subsequent tunneling through the triangle barrier (Figure 4b). As a result, an additional PC peak on the low energy side of the spectra appears.

**Figure 5:**
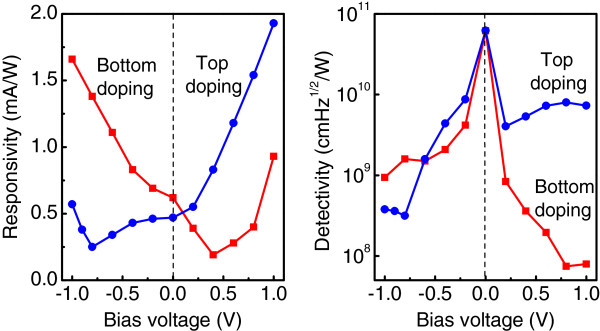
**Measured responsivity and detectivity as a function of applied bias.** For Ge/Si QDIPs whose PC spectra are presented in Figures 2 and 3. For the sample with bottom doping, the data were taken at *λ *= 3 *μ*m. For the sample with top doping, the peak responsivity and detectivity (*λ *= 3.4 *μ*m) are shown.

The measured responsivity and detectivity of the two QDIPs with bottom and top positions of the doping planes are depicted in Figure 5. For both samples we find that at *U*_*b *_= 0 V, the measured noise current is closed to the calculated thermal noise. This leads to a better performance when detectors are operating in the photovoltaic regime, where specific detectivity reaches the value of *D*^⋆ ^= 6.2 × 10^10^ cm Hz^1/2^/W at *T *= 90 K, with a corresponding responsivity of 0.5 to 0.6 mA/W. The important observation is a reversal of the voltage dependence of responsivity with respect to zero bias when the *δ*-doping plane is moved from the bottom to the top of the dot layer. This result undoubtedly points out that the main reason for the asymmetric photoresponse is the existence of a built-in electric field due to the charged *δ*-doping plane and is not caused by the asymmetry of the strain distribution, dot shape, and wetting layer as proposed in [12,13].

**Figure 6:**
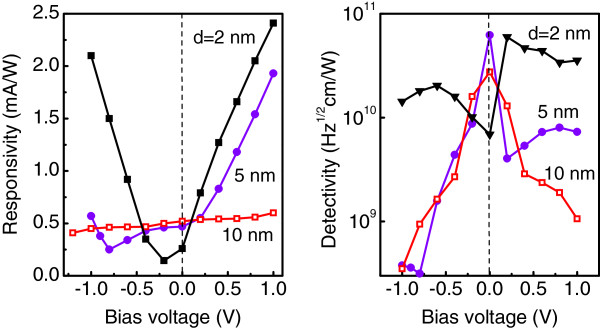
**Measured responsivity and detectivity.** As a function of applied bias for Ge/Si QDIPs in which the distance between the *δ*-doping layer and the QD plane *d* was varied from 2 to 10 nm. All devices have a top doping configuration with *p*_B _= 8 × 10^11^cm^−2^.

Figure 6 displays the bias dependent responsivity and detectivity from three detectors in which the distance between the *δ*-doping layer and the QD plane was varied from 2 to 10 nm. The most symmetrical *R*(*U*_*b*_) and *D*^⋆^(*U*_*b*_) characteristics and the less pronounced photovoltaic effect are realized in a device with *d *= 2 nm (the peak wavelength is 3.5 *μ*m). For this sample, the highest detectivity of 6.0 × 10^10^ cm Hz^1/2^/W is observed at *U*_*b *_= 0.2 V, i.e., in a photoconductive mode. A possible reason responsible for the suppression of the photovoltaic effect at small *d* is the penetration of boron atoms into the QD layer, which results in a more symmetric electric-field distribution around the dots.

**Figure 7:**
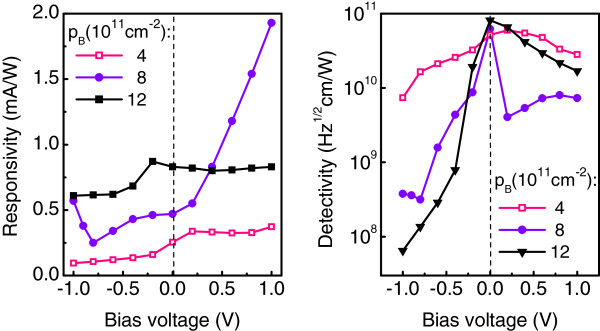
**Measured responsivity and detectivity.** As a function of applied bias for Ge/Si QDIPs in which the sheet boron density in the *δ*-doping layers was varied from 4 × 10^11 ^to 12 × 10^11^cm^−2^. All devices have a top doping configuration with *d *= 5 nm.

Figure 7 shows the responsivity and detectivity for three detectors in which the sheet boron density in the *δ*-doping layers was varied from 4 × 10^11^ to 12 × 10^11^ cm^−2^. The distance between the dots and the doping plane was *d *= 5 nm. An increase of doping density leads to a higher responsivity at *U*_*b *_< 0.4 V. This is likely in agreement with the theoretical prediction [14]. The best performance is achieved for *p*_B _= 12 × 10^11^cm^−2^ in the photovoltaic mode, where *R *= 0.83 mA/W and *D*^⋆ ^= 8.1 × 10^10^ cm Hz^1/2^/W at *λ *= 3.4 *μ*m. The highest peak detectivity of the sample with a lower doping density is observed in the photoconductive mode at *U*_*b *_= 0.2 V.

## Conclusions

We have presented the performance characteristics of Ge/Si(001) quantum dot mid-infrared detectors in which the capping temperature *T*_cap _and parameters of doping were systematically varied. With increasing *T*_cap _from 300°C to 750°C, the photocurrent peak wavelength shifts from 2.3 to 3.9 *μ*m, reflecting enhanced Si-Ge intermixing at higher overgrowth temperatures. The devices with lower doping density or shorter QD-dopant separation have better performance in a photoconductive mode while other detectors display the highest specific detectivity at zero bias. We demonstrated that the main reason for the photovoltaic response is the existence of a built-in electric field due to the charge redistribution between QDs and *δ*-doping layers. The highest photovoltaic detectivity and responsivity at 90 K are *D*^⋆ ^= 8.1 × 10^10^ cm Hz^1/2^/W and *R *= 0.83 mA/W, respectively, for *T*_cap _= 600°C and the 3.4-*μ*m photoresponse peak. The possibility of the device to operate in a photovoltaic mode makes it compatible with existing Si-readout circuits in focal plane array applications.

## Abbreviations

λ: photon wavelength; ML: monolayer; PC: photocurrent; QD: quantum dot; QDIP: quantum dot infrared photodetector.

## Competing interests

The authors declare that they have no competing interests.

## Authors’ contributions

AY conceived and designed the experiment, carried out the photocurrent measurements, participated in the discussions, and drafted the manuscript. VT and AN prepared the samples using molecular beam epitaxy technique. AB performed the noise measurements. AD supervised the project. All authors read and approved the final manuscript.

## Authors’ information

AY is a leading researcher in the Institute of Semiconductor Physics. His research interests are in the area of electronic processes in low-dimensional systems. VT is a postgraduate student working on nanotechnology. AB is a researcher specializing in computational simulation physics. AN is the head of the Laboratory of Molecular Beam Epitaxy. Current research topics in his group include growth process studies of Ge quantum dots on Si, delta-doped Si layers, and low-temperature growth. AD is a professor and a member of the scientific boards on the problems of Semiconductor Physics and Radiation Physics of Solid State in Russian Academy of Science.
